# Honest sexual signalling mediated by parasite and testosterone effects on oxidative balance

**DOI:** 10.1098/rspb.2008.1570

**Published:** 2008-12-02

**Authors:** Francois Mougeot, Jesu´s Martínez-Padilla, Lucy M.I. Webster, Jonathan D. Blount, Lorenzo Pérez-Rodríguez, Stuart B. Piertney

**Affiliations:** 1 Estación Experimental de Zonas Áridas, CSICc. General Segura 1, 04001 Almeria, Spain; 2 Instituto de Investigación en Recursos Cinegéticos, IREC (CSIC, UCLM, JCCM)Ronda de Toledo s/n, 13005 Ciudad Real, Spain; 3 School of Biological Sciences, University of AberdeenAberdeen AB24 2TZ, UK; 4 Centre for Ecology & Conservation, School of Biosciences, University of ExeterCornwall Campus, Penryn TR10 9EZ, UK

**Keywords:** oxidative stress, antioxidant, ornament, trade-off, red grouse *Lagopus lagopus scoticus*, *Trichostrongylus tenuis*

## Abstract

Extravagant ornaments evolved to advertise their bearers' quality, the honesty of the signal being ensured by the cost paid to produce or maintain it. The oxidation handicap hypothesis (OHH) proposes that a main cost of testosterone-dependent ornamentation is oxidative stress, a condition whereby the production of reactive oxygen and nitrogen species (ROS/RNS) overwhelms the capacity of antioxidant defences. ROS/RNS are unstable, very reactive by-products of normal metabolic processes that can cause extensive damage to key biomolecules (cellular proteins, lipids and DNA). Oxidative stress has been implicated in the aetiology of many diseases and could link ornamentation and genetic variation in fitness-related traits. We tested the OHH in a free-living bird, the red grouse. We show that elevated testosterone enhanced ornamentation and increased circulating antioxidant levels, but caused oxidative damage. Males with smaller ornaments suffered more oxidative damage than those with larger ornaments when forced to increase testosterone levels, consistent with a handicap mechanism. Parasites depleted antioxidant defences, caused oxidative damage and reduced ornament expression. Oxidative damage extent and the ability of males to increase antioxidant defences also explained the impacts of testosterone and parasites on ornamentation within treatment groups. Because oxidative stress is intimately linked to immune function, parasite resistance and fitness, it provides a reliable currency in the trade-off between individual health and ornamentation. The costs induced by oxidative stress can apply to a wide range of signals, which are testosterone-dependent or coloured by pigments with antioxidant properties.

## 1. Introduction

Testosterone plays a pivotal role in regulating the expression of many animal ornaments (Folstad & Karter 1992; Wingfield *et al.* 2001). Males typically benefit from elevated testosterone levels and enhanced ornamentation in intra- and inter-sexual contexts, but maintaining high testosterone levels may be costly (Folstad & Karter 1992). A main cost might be a reduced ability to resist parasites: according to the immunocompetence handicap hypothesis (ICHH) testosterone impairs immune function, so only individuals of high genetic quality can endure the cost of displaying larger ornaments (Folstad & Karter 1992). The idea that testosterone may suppress the immune system in birds has, however, received mixed support (Roberts *et al.* 2004), possibly because the costs are not entirely mediated through the physiological pathways hitherto examined (Owen-Ashley *et al.* 2004; Mougeot *et al.* 2005*b*; Blas *et al.* 2006). The oxidation handicap hypothesis (OHH), a refinement of the ICHH, proposes that the trade-off ensuring honest signalling is between ornamentation and oxidative stress (von Schantz *et al.* 1999; Alonso-Alvarez *et al.* 2007). Elevated testosterone can lead to increased reactive oxygen and nitrogen species (ROS/RNS) production and oxidative stress (Alonso-Alvarez *et al.* 2007), which may, in turn, impair lymphocyte proliferation and signalling pathways involved in an immune response (Larbi *et al.* 2007). Additionally, immune system activation produces ROS/RNS to help counter invading pathogens (Romero *et al.* 1998; Hõrak *et al.* 2007), but their overproduction can lead to oxidative stress, incurring damage to host tissues including ornaments, particularly when individuals lack sufficient antioxidant protection (von Schantz *et al.* 1999; Splettstoesser & Schuff-Werner 2002; Halliwell & Gutteridge 2007). Testosterone-dependent ornaments may thus be inherently vulnerable to oxidative stress, itself intimately linked to immune function and parasite resistance (von Schantz *et al.* 1999). The OHH therefore proposes that only high-quality individuals, with a prime antioxidant system, could afford the costs (increased oxidative stress) of maintaining high testosterone levels and enhanced ornamentation.

We tested the OHH in free-living red grouse (*Lagopus lagopus scoticus*). This bird displays supra-orbital red combs, the coloration of which is carotenoid based (Mougeot *et al.* 2007*a*,*b*) and whose size is testosterone dependent (Mougeot *et al.* 2005*a*). Comb size functions in intra- and inter-sexual selection: males with higher testosterone levels and bigger combs benefit by being dominant, more aggressive, holding larger territories and being more attractive to females (Mougeot *et al.* 2003; Redpath *et al.* 2006). Bigger combs are often, but not always, redder, and seem likely to contain absolutely more carotenoids although this remains to be tested. Therefore, the possibility exists that the comb size and comb colour reveal similar information about individual quality. However, the signalling function of comb colour remains untested, so we focus our study on comb size. Using a factorial experimental design, we manipulated (i) parasite burdens (P) of the nematode *Trichostrongylus tenuis*, which has well-known negative effects on this host (Hudson 1986; Delahay *et al.* 1995), using experimental infections (Hudson 1986; Delahay *et al.* 1995; Mougeot *et al.* 2005*b*) and (ii) testosterone levels (T) using implants (Mougeot *et al.* 2005*a*). We initially purged males of *T. tenuis* (Hudson 1986) and began manipulations 15 days later. We then randomly assigned males to one of four treatment groups (10 in each): (i) empty implants, no parasite challenge (T−P− males; control group), (ii) empty implants, challenge with *T. tenuis* infective larvae (T−P+ males), (iii) testosterone implants, no parasite challenge (T+P− males), and (iv) testosterone implants, challenge with *T. tenuis* (T+P+ males). We sampled males before treatments (S1) and again after 10 days (S2) and 17 days (S3; see appendix A). We investigated treatment effects on (i) plasma testosterone concentration, (ii) *T. tenuis* abundance, (iii) ornamentation (comb area), (iv) total antioxidant status (TAS; an index of circulating antioxidant defences) and (v) plasma concentrations of malondialdehyde (MDA), a measure of oxidative damage. TAS measures the capacity of the plasma to quench a free radical cation and the pooled effect of all extracellular, non-enzymatic antioxidants in plasma (e.g. uric acid, vitamins C and E, carotenoids) (von Schantz *et al.* 1999; Halliwell & Gutteridge 2007). MDA is formed when lipid hydroperoxides break down, a process (lipid peroxidation) caused by oxidative stress (Romero *et al.* 1998).

We predicted that (i) increased testosterone levels would enhance ornamentation but increase oxidative damage, measured in terms of MDA, (ii) a developing parasite infection would reduce circulating antioxidants, cause oxidative damage and reduced ornamentation, (iii) the cost of each treatment (oxidative damage) would depend on initial ornament size, with males displaying smaller combs showing a greater increase in oxidative damage (MDA) relative to larger combed males, and (iv) the treatment effects on oxidative damage and the ability of males to increase antioxidant activity (TAS) to reduce this damage would explain changes in male ornamentation.

## 2. Material and methods

### (a) Experiment

We worked on Edinglassie and Catterick moors (UK) in 2006. In September, we caught 20 male red grouse on each site, by dazzling and netting them at night (Hudson 1986). Upon first capture (S0; see appendix A), we fitted males a radio-collar (TW3-necklace radio-tags, Biotrack) and gave each a 1 ml of levamisole hydrochloride (Nilverm Gold, Schering-Plough Animal Health, Welwyn Garden City, UK) to purge them of their *T. tenuis* nematodes (Hudson 1986). We started the experiment 15 days later, allowing birds enough time to clear the anthelminthic. At S1, we gave males hormone and parasite treatments (five males per treatment per site). Males were implanted with two silastic tubes (each 20 mm long, 1.57 mm inner and 2.41 mm outer diameter) sealed with glue at both ends. T− males were given two empty implants, and T+ males two implants filled with crystalline testosterone propionate (Sigma Aldrich, UK) to elevate testosterone for two to three months (Mougeot *et al.* 2003). Implants were inserted subcutaneously on the flank following local anaesthesia. P+ males received an oral dose of water containing approximately 5000 *T. tenuis* infective larvae and P− males only water. We sampled males upon treatment (S1), 10 days later (S2) and 17 days later (S3). Details on the timing and data sampling for the experiment are given in appendix A. We held all the necessary Home Office licences for conducting the procedures described in this work (Licence PPL80/1437).

### (b) Measurements and blood sampling

We measured comb area (maximum length×width of flattened comb) as an index of ornament size (Mougeot *et al.* 2005*a*). We took a blood sample from the brachial vein, separated plasma by centrifugation (2 min at 7000 rpm) and froze the samples in liquid nitrogen within 5 min of collection. Plasma samples were taken to the laboratory afterwards and stored at −80°C.

### (c) Testosterone assays

Plasma testosterone concentration was measured using a commercially available testosterone enzyme immunoassay (Elisa Kit EIA-1559 from DRG Diagnostics, Marburg, Germany), an assay that has been developed and validated for determining testosterone levels in small volume (20 μl) avian plasma samples (Washburn *et al.* 2007). Intra- and interassay coefficients of variation were 3.59 and 7.14 per cent, respectively, and the detection limit was 0.2 ng ml^−1^. Repeatability was determined on a subsample measured twice (*r*=0.88; *n*=30, *p*<0.001).

### (d) Lipid peroxidation assays

Plasma concentrations of MDA were calculated by HPLC using fluorescence detection (Agarwal & Chase 2002). All chemicals were HPLC grade, and chemical solutions were prepared using ultra pure water (Milli-Q Synthesis; Millipore, Watford, UK). Assays were carried out in 2 ml capacity screw-top microcentrifuge tubes. To a 15 μl aliquot of sample or standard (1,1,3,3-tetraethoxypropane, TEP; see below), 15 μl butylated hydroxytoluene solution (0.05% w/v in 95% ethanol), 120 μl phosphoric acid solution (0.44 M) and 30 μl thiobarbituric acid solution (42 mM) were added. Samples were capped, vortex mixed for 5 s, then heated at 100°C for 1 h on a dry bath incubator to allow formation of MDA-TBA adducts. Samples were then cooled on ice for 5 min, before 75 μl *n*-butanol was added and tubes were vortex mixed for 30 s. Tubes were then centrifuged at 12 000× min^−1^ and 4°C for 3 min, before a 50 μl aliquot of the upper (*n*-butanol) phase was collected and transferred into an HPLC vial for analysis. Samples (10 μl) were injected into a Dionex HPLC system (Dionex Corporation, California, USA) fitted with a 5 μm Octadecyl-silica (ODS) guard column and a Hewlett-Packard Hypersil 5 μm ODS 100×4.6 mm column maintained at 37°C in a thermostatted column compartment (TCC-100; Dionex). The mobile phase was methanol buffer (40 : 60, v/v), the buffer being a 50 mM anhydrous solution of potassium monobasic phosphate at pH 6.8 (adjusted using 5 M potassium hydroxide solution), running isocratically over 3.5 min at a flow rate of 1 ml min^−1^. The data were collected using a fluorescence detector (RF2000; Dionex) set at 515 nm (excitation) and 553 nm (emission). For calibration, a standard curve was prepared using a TEP stock solution (3 μM in 40% ethanol) serially diluted using 40 per cent ethanol. TEP standards assayed in duplicate showed high repeatability (*r*=0.996, *n*=11, *p*<0.0001).

### (e) Total antioxidant status

TAS concentration of plasma was assessed by means of commercial kits (Randox Laboratories Ltd, Crumlin, UK) adapted to an automated spectrophotometer (A25-Autoanalyzer; Biosystems SA, Barcelona). Plasma samples were incubated for 15 s with a chromogen composed of metmyoglobin and ABTS (2,2-azino-di-[3-ethylbenzthiazoline sulphonate]). Hydrogen peroxide (H_2_O_2_) was then added and the sample was incubated for 195 s. Hydrogen peroxide (H_2_O_2_) addition induces the production of the radical cation ABTS, which generates a blue-green colour. Colour is measured at 600 nm before and after H_2_O_2_ addition, thus determining the change in colour. Antioxidants in the plasma sample cause suppression of this colour change to a degree that is proportional to their concentration. Results are given as mmol l^−1^ of plasma. Repeatability was determined on a subsample measured twice (*r*=0.92, *n*=30, *p*<0.001).

### (f) Parasite counts, cultures and challenges

We estimated *T. tenuis* abundance using either caecal egg counts (from caecal samples collected from captured males at S1) or direct worm counts (from caeca collected from males humanly killed at the end of the experiment, at S3). Caecal egg counts provide reliable estimates of worm burdens and were used to calculate *T. tenuis* abundance (Seivwright *et al.* 2004). More details regarding the methods for estimating *T. tenuis* abundance from caecal egg counts or direct worm counts, and for cultivating larvae for challenges are given elsewhere (Shaw 1988; Moss *et al.* 1990; Seivwright *et al.* 2004; Mougeot *et al.* 2005*b*).

### (g) Statistical analyses

We used SAS v. 8.01 (SAS 2001). Counts of *T. tenuis* worms were fitted to generalized linear models using a Poisson error distribution. We calculated individual changes over time in study parameters (testosterone, comb area, MDA and TAS) as the difference between the final and initial values, corrected for the initial value (residuals from a general linear model). We tested whether these changes over time differed according to testosterone treatment (TTreat), parasite treatment (PTreat) and their interaction using general linear models. All models also included ‘site’ as a fixed effect to control for possible differences between sites. Experimental results did not differ between sites.

## 3. Results

**Figure 1:**
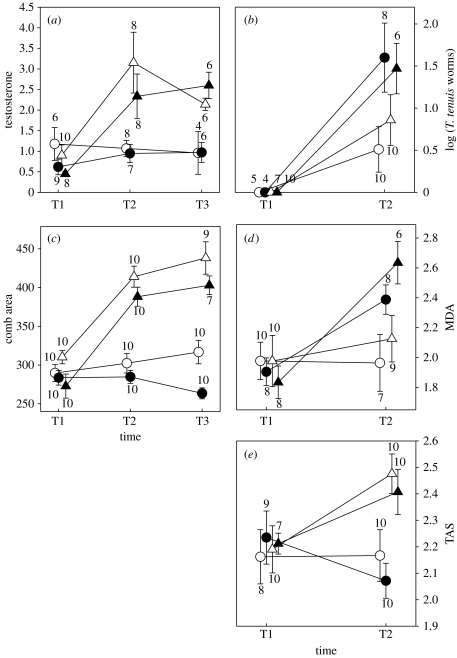
Effects of hormone and parasite treatments on changes over time in mean±s.d. (*a*) Plasma concentration of testosterone (ng ml^−1^), (*b*) *T. tenuis* nematode abundance (log-transformed number of worms per male), (*c*) comb area (mm^2^), (*d*) plasma concentration of MDA (nmol ml^−1^) and (*e*) TAS (mmol ml^−1^). S1, first sampling (immediately prior to treatments); S2 and S3, subsequent samplings, 10 and 17 days later. Sample sizes refer to the number of males. Open circles, T−P− males; filled circles, T−P+ males; open triangles, T+P− males; filled triangles, T+P+ males.

At the start of the experiment (S1), males that had been dosed previously with anthelminthic (at S0) had no *T. tenuis* eggs in their faeces (*n*=5, 4, 7 and 10 males sampled in the T−P−, T+P−, T−P+ and T+P+, respectively), indicating that the initial parasite purging had been effective. Testosterone levels increased more in T+ than in T− males between S1 and S2 (table 1; figure 1*a*) and remained higher afterwards (S2 until S3). Parasite challenges had no effect on testosterone levels (table 1; figure 1*a*), but increased *T. tenuis* abundance. When challenged (S1), males had no detectable *T. tenuis* worms. However, by S3, average *T. tenuis* abundance was higher in P+ (mean 147 worms) than in P− males (15 worms), irrespective of the testosterone treatment (figure 1*b*; PTreat: *F*_1,25_=10.57, *p*=0.001; TTreat: *F*_1,25_=0.46, *p*=0.49; Ptreat×TTreat: *F*_1,25_=0.10, *p*=0.74).

Consistent with prediction 1, testosterone implants increased comb area and the concentration of MDA. Comb area increased more in T+ than in T− males between S1 and S2 and remained larger afterwards (table 1; figure 1*c*). MDA concentration increased more in T+ than in T− males (table 1; figure 1*d*).

Consistent with prediction 2, parasite challenge caused oxidative damage (MDA concentration increased more in P+ than in P− males; table 1; figure 1*d*). Parasite challenge reduced ornamentation, but only in T− males and with a time lag (comb area decreased in T−P+ males while it increased in T−P− males between S2 and S3; table 1; figure 1*c*).

Consistent with prediction 3, changes in MDA were dependent on initial comb area, but in T+ males only. In T+ males, changes in MDA were not explained by parasite treatment, but by initial comb area (Ptreat: *F*_1,14_=0.77, *p*=0.39; comb: *F*_1,14_=4.68, *p*<0.05; slope: −0.004±0.002). In T− males, changes in MDA were explained by parasite treatment, irrespective of initial comb area (GLM: Ptreat: *F*_1,13_=7.36, *p*<0.05; comb: *F*_1,13_=0.90, *p*=0.23; slope ±s.d.: +0.002±0.002).

TAS increased more in T+ than in T− males, while parasite treatment reduced TAS, but depending on testosterone treatment (table 1). In T+ males, elevated testosterone caused an increase in the levels of circulating antioxidant defences, irrespective of parasite treatment (PTreat: *F*_1,14_=0.11, *p*=0.74; figure 1*e*). In T− males, parasite treatment reduced TAS (*F*_1,14_=8.09; *p*<0.05; figure 1*e*).

**Figure 2:**
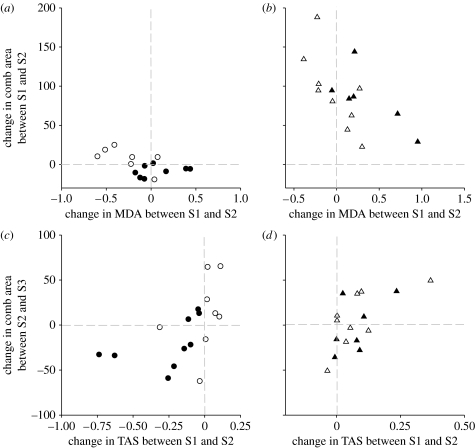
Changes in ornament size (comb area mm^2^) between sampling times (S1–S2 and S2–S3) according to the treatments and to (*a*,*b*) oxidative damage extent (changes in MDA nmol ml^−1^) or (*c*,*d*) changes in circulating antioxidant defences (TAS nmol ml^−1^). Symbol descriptions are the same as in the legend of figure 1.

Before testosterone and parasite treatments (at S1), MDA was not significantly related to TAS (*F*_1,30_=2.41, *p*=0.13; slope ±s.d.: +0.499±0.323). Changes in MDA between S1 and S2 were not significantly related to changes in TAS (*F*_1,29_=1.22, *p*=0.28; slope ±s.d.: +0.404±0.365). However, changes in TAS and MDA explained the impact of treatments on changes in comb area after taking into account treatment group level effects (table 2). Changes in MDA explained the extent to which testosterone implants initially increased ornamentation (between S1 and S2), depending on testosterone treatment (table 2; significant ΔMDA×TTreat interaction). In T+ males, individuals that increased their comb area most were least susceptible to oxidative stress (*F*_1,11_=10.09, *p*<0.01; figure 2*b*), while no such effect was found in T− males (*F*_1,10_=0.24, *p*=0.63; figure 1*a*).

Consistent with prediction 4, changes in TAS explained lagged changes in ornamentation, between S2 and S3 (table 2; figure 2*c*,*d*). In T− males, a reduction in TAS was associated with a decrease in ornament size (figure 2*c*). In T+ males, a greater increase in TAS was associated with a continued increase in ornamentation (figure 2*d*).

## 4. Discussion

Testosterone treatment successfully increased testosterone levels, which were higher in T+ than in T− males after implantation (S2 and S3), but were still within the natural range (Mougeot *et al.* 2005*a*). Parasite treatment also successfully increased *T. tenuis* infection levels (higher in P+ than in P− males at S3). There was no short-term effect of testosterone on the effectiveness of parasite challenges, although previous work showed that elevated testosterone can indirectly increase *T. tenuis* abundance 1 year after challenge (Mougeot *et al.* 2005*b*; Seivwright *et al.* 2005).

Testosterone implants enhanced ornamentation (prediction 1), while parasite challenges reduced ornamentation (prediction 2), but only in T− males, and with a time lag. *Trichostrongylus tenuis* larvae impact most on metabolism 12–16 days after infection (Delahay *et al.* 1995) explaining this delayed effect. The parasite challenges did not reduce ornamentation in T+ males. This may be because the implants forced males to circulate high testosterone levels, which were similar in T+P− and T+P+ males, such that the exogenous testosterone would have prevented a possible parasite-induced reduction in testosterone levels and ornamentation.

Testosterone implants increased oxidative damage, as indexed by MDA concentrations (prediction 1). This might be because testosterone increased metabolic rates (e.g. Buchanan *et al.* 2001), or impaired the activity of antioxidant defences (e.g. Alonso-Alvarez *et al.* 2007), thereby increasing the oxidative stress. Smaller combed males suffered more oxidative damage (greater increase in MDA) than larger combed males when forced to circulate testosterone levels above their individual optima (T+ males only). This is consistent with a handicap mechanism (prediction 3), where the cost of testosterone (increased oxidative damage) would be greater for males with lesser ornamentation and a lower testosterone optimum (Zahavi 1975; Folstad & Karter 1992; Getty 2002).

Interestingly, elevated testosterone resulted in an increase in antioxidant defences as measured by the TAS assay *in vitro*, suggesting that the antioxidant defences were upregulated *in vivo*. However, this was not sufficient to prevent oxidative damage. Parasite challenges also increased oxidative damage (prediction 2) but reduced TAS when these were not increased by testosterone (in T−P+ as compared with T−P− males). Overall, parasite challenge caused more oxidative damage than experimental testosterone increase, possibly because of the contrasted effects of these manipulations on circulating antioxidant defences.

Testosterone can pose an oxidative challenge, which can be controlled by increasing investment in antioxidant defences (allocating resources towards self maintenance) but at the cost of investing these same resources to sexual signal expression. Such a trade-off was supported for carotenoid-based coloured traits controlled by testosterone: elevated testosterone may increase or decrease signal coloration in a direct trade-off with circulating plasma carotenoids (Alonso-Alvarez *et al.* 2008). In our experiment, testosterone implants increased in TAS. This effect could have arisen because testosterone invoked greater foraging effort by individuals, or directly enhanced the assimilation of dietary antioxidants, such as carotenoids and vitamin E, by upregulating the synthesis of lipoproteins that are responsible for transporting these compounds (McGraw *et al.* 2006). Experimentally elevated testosterone can cause increased plasma levels of carotenoids, as found in several birds (Blas *et al.* 2006; McGraw & Ardia 2007; Alonso-Alvarez *et al.* 2008). In previous experiments on red grouse, testosterone enhanced both comb size and carotenoid-based comb colour, most likely by causing a short-term increase in circulating carotenoids, even if testosterone implanted males did not circulate higher carotenoid levels than control males after a month (Mougeot *et al.* 2007*b*). The reduction in TAS caused by parasites could also be explained by a reduction in circulating carotenoids (*T. tenuis* parasites were shown by experiment to reduce circulating carotenoids: Martinez-Padilla *et al.* 2007; Mougeot *et al.* 2007*b*), and also possibly because parasite challenges reduced comb area and smaller ornaments would require fewer carotenoids for pigmentation. However, whether carotenoid pigments act as antioxidants in birds *in vivo* has been both supported and questioned, and remains controversial (Costantini & Møller 2008). Clearly, more work is needed to better understand how testosterone can increase TAS, and the contribution of carotenoids to circulating antioxidant defences.

Our experiment showed that two key factors influencing ornamentation, testosterone and parasites, additively caused oxidative damage. Moreover, the impacts of testosterone and parasites on oxidative balance (changes in circulating antioxidants and oxidative damage) explained short- and medium-term treatment effects on male ornamentation, showing for the first time that the ability to express a testosterone-dependent ornament is tightly related to an individual's oxidative balance and susceptibility to oxidative stress. The extent to which testosterone and parasites cause oxidative damage depends on an individual's ability to increase circulating antioxidant defences (by acquiring more or mobilizing stored antioxidants) and to resist parasites (the ability of its activated immune system to raise an appropriate immune response that finds the right target and at the same time avoid immunopathological damage; Råberg *et al.* 1998; von Schantz *et al.* 1999). Antioxidants are depleted during immune responses (von Schantz *et al.* 1999), while increasing circulating antioxidants can reduce the negative impact of ROS/RNS on immune responses (Blas *et al.* 2006; Larbi *et al.* 2007). The increase in circulating antioxidants caused by testosterone might have buffered the impact of parasite challenges on ornamentation, and could explain why changes in comb area did not differ between T+P− and T+P+ males.

Ornaments have evolved to facilitate the assessment of individual quality (Andersson 1994), such as heritable parasite resistance (Hamilton & Zuk 1982). Immune and detoxification systems identify foreign compounds and destroy pathogens or excrete toxic substances, but their activation often generates ROS/RNS and induces oxidative stress (von Schantz *et al.* 1999; Finkel & Holbrook 2000). A main advantage of ornaments that reflect susceptibility to oxidative stress is that the cost can be directly related to an individual's heritable ability to resist pathogens and toxic compounds (von Schantz *et al.* 1999). The OHH (von Schantz *et al.* 1999; Alonso-Alvarez *et al.* 2007) provides a potent refinement of the ICHH (Folstad & Karter 1992); considering oxidative stress as pivotal in the trade-off between immune function and ornamentation provides an alternative pathway to testosterone-induced immunosuppression (Owen-Ashley *et al.* 2004; Mougeot *et al.* 2005*b*), and should help explain discrepancies found among studies and species (Roberts *et al.* 2004). Another advantage is that oxidative stress provides a reliable cost for honest signalling that applies to a wide range of common animal ornaments, whose expression is testosterone dependent (Folstad & Karter 1992) or that are coloured by pigments with antioxidant properties (McGraw 2005; Blas *et al.* 2006; Galván & Alonso-Alvarez 2008).

## Figures and Tables

**Table 1:** Effect of testosterone and parasite treatments on changes over time in testosterone concentration (Δtestosterone), ornamentation (Δcomb area), plasma concentration of malondialdehyde (ΔMDA) and total antioxidant status (ΔTAS).

treatment effects:			TTreat^a^		PTreat^b^		TTreat×PTreat	
					
dependent:	sampling time^c^	d.f.	*F*	*p*-value	*F*	*p*-value	*F*	*p*-value
Δtestosterone^d^	S1–S2	1,22	17.76	<0.001	0	0.96	0.92	0.35
Δtestosterone^d^	S2–S3	1,11	3.59	0.08	0.1	0.75	0.56	0.47
Δcomb area^d^	S1–S2	1,32	80.01	<0.001	0.27	0.61	0.16	0.7
Δcomb area^d^	S2–S3	1,30	0.95	0.34	1.91	0.17	2.7	<0.05
ΔMDA^d^	S1–S2	1,28	5.32	<0.05	10.88	<0.01	0.01	0.92
ΔTAS^d^	S1–S2	1,31	16.1	<0.001	4.31	<0.05	3.41	0.07

a TTreat=testosterone treatment: T− males, sham implanted; T+ males, implanted with testosterone.

b PTreat=parasite treatment: P− males, not challenged; P+ males, challenged with infective *T. tenuis* larvae.

c S1=first sampling (day 0); S2=second sampling (day 10), S3=third sampling (day 17). MDA and TAS were measured at S1 and S2 only.

d Changes in study parameters were calculated as the difference between the final and initial values corrected for initial values. MDA and TAS were measured at S1 and S2 only.

**Table 2:** Effect of treatments, changes in plasma concentration of malondialdehyde (ΔMDA) and antioxidant activity (ΔTAS) on changes in ornamentation (Δcomb) at different sampling times (S1–S2; S2–S3).

		Δcomb^a^ S1–S2		Δcomb^a^ S2–S3	
			
dependent variable:	d.f.	*F*	*p*-value	*F*	*p*-value
TTreat^b^	1,19	84.12	<0.001	0	0.96
PTreat^c^	1,19	1.09	0.31	4.98	<0.05
TTreat×PTreat	1,19	0.06	0.8	3.19	0.09
ΔMDA^d^	1,19	13.07	<0.01	1.29	0.27
ΔTAS^e^	1,19	0.46	0.51	8.54	<0.01
ΔMDA×TTreat	1,19	4.09	<0.05	0.82	0.37
ΔTAS×TTreat	1,19	0.39	0.54	1.12	0.29
ΔMDA×PTreat	1,19	2.62	0.12	3.16	0.09
ΔTAS×PTreat	1,19	0.02	0.88	0.3	0.59

a Δcomb was calculated as the difference between the final and initial comb area, corrected for the initial comb area.

b TTreat=males, sham implanted (T−) versus testosterone implanted (T+) males.

c PTreat=parasite treatment, categorical: P− males: not challenged; P+ males: challenged with infective *T. tenuis* larvae.

d ΔMDA was calculated as the difference in MDA concentration between S2 and S1 corrected for MDA concentration at S1.

e ΔTAS was calculated as the difference in TAS concentration between S2 and S1 corrected for TAS concentration at S1.

**Table tbl3:** 

events	initial capture	first sampling (S1)	second sampling (S2)	third sampling (S3)
dates	25 Sep ±5 days	10 Oct ±5 days	20 Oct ±5 days	27 Oct ±2 days
procedures	Purging of *T. tenuis* worms	experiment start		experiment end
		hormone implants		
		parasite challenges		
measurements^a^		*T. tenuis* parasites		*T. tenuis* parasites
		testosterone	testosterone	testosterone
		comb area	comb area	comb area
		MDA	MDA	
		TAS	TAS	

a We measured parasites at S1 (using faecal samples) and S3 (using direct worm counts) to check that the parasite purging conducted at S0 had been effective (S1) and to check that parasite challenges were effective (S3), respectively. We did not measure parasites at S2 because it takes 10–15 days for *T. tenuis* larvae to develop into measurable, egg-producing worms using faecal samples. Not all parameters (parasites, testosterone, MDA and TAS) could be measured for all individuals at each sampling time, due to lack of sample material (faecal samples or plasma), so sample size varies between treatment groups and sampling times.

## Appendix A Timing of the experiment, procedures and data sampling

[Table tbl3]
